# The Modern London Training-School for Nurses

**Published:** 1919-05-24

**Authors:** 


### THE MATRONS' AND SISTERS' DEPARTMENT.

### THE MODERN LONDON TRAINING-SCHOOL FOR NURSES.

At the moment, three London hospitals with medical schools—St. Bartholomew's, the Middlesex, and St. George's—are getting out plans and collecting funds for the building of new Nurses' Homes, while the Edith Cavell Home at the London Hospital has recently been opened. Collectively, the sums required and contributed for the housing of probationers and sisters in London alone run to something like a million and a half. It is natural to inquire what the public and the nurses are expected to get from this vast expenditure.

There are two schools of opinion in the matter of nurses' quarters. A small, but noisy, minority demand that the Nurses' Homes in connection with hospitals shall be hostels pure and simple, boarding-houses for the convenience of those employed at the hospital. They would remove all restrictions and personal control over the inmates, permit the nurses to come and go as they wished and avoid like poison any attempt to exercise influence.

The other and saner school of opinion holds that the Nurses' Home is a collegiate establishment, and that nurses in training should enjoy special opportunities of enlarging their views upon life and enriching their characters at the same time that they are acquiring the principles and practice of the nursing art. This second theory has never yet been carried out to its full extent, but new foundations give ground for hope that it may be realised in the future. Take them whence you will, young women are but immature creatures when they begin their training.

Is it true that many women emerge from their training a little vulgarised, a little narrower, less interested in the world of art, letters, music, and science, than when they first entered the portals of their hospital? We hold that unless steps be taken to guard them from the wrong associations and introduce them to higher ideals this must necessarily be the case. How many women come up from the country to be trained and leave London utterly unaware of the strong currents of intellectual life which move beneath the paltry shops and amusements level? Is it well to leave the probationer alone amidst what may be called the scum of London life when she might, by wise guidance, find it a storehouse of all that is greatest and most enduring in the national life?

The aim of the London Training-School should stretch far beyond the desire to attract good candidates for training by sumptuous rooms and attention to their comfort and convenience. The lecture-room should be ample to serve the needs of any group of affiliated hospitals which may be assigned to it when the profession is organised. Lectures, as at present arranged, are often too scantily attended to stimulate the busy surgeon or physician to give of his best. They are often perfunctory, and, truth to tell, either childish or obscure. By collecting in the hall of the central training-schools, north, south, east, and west, probationers from every neighbouring institution this blot could be wiped out, for adequate fees would command adequate services. Possibly, tutorial help from the centre might be available for the pupils from the smaller establishments. Thus learning and enthusiasm could be introduced where now dulness and mediocrity are suffered to reign.

But in the collegiate establishment under contemplation much more could be done. Lecturers on outside subjects could be invited in accordance with some varied curriculum of extra professional training. Periodical expeditions to places of interest could be organised, for there are many curators who would arrange a specially interesting visit for the nurses to museum, or art gallery, or monument of antiquity, so that they came as students, not as sightseers. Groups of music-lovers might, by co-operation, obtain special consideration, cheap seats at classical concerts being reserved always for nurses unable to wait in a queue. The nurses' library, too, should admit new books, not necessarily novels, and some encouragement to reading should be afforded, while a music library should not be forgotten. And thus the foundations would be laid for a live modern debating society, in which the nurses might learn to express themselves, and prepare by contact with matters of intellectual value for the intelligent government of their own profession.

What nurses in training need is to have the latent forces of their nature drawn out and stimulated, not always in one groove. The London Training-Schools have unique opportunities for developing the broad-minded, well-balanced nursing sisters needed for the world's work, and in planning their Nurses' Homes such aims should be kept in view.

